# High-Resolution Optical Chromatography: Principles, Innovations, and Emerging Biomedical Applications

**DOI:** 10.3390/mi17060661

**Published:** 2026-05-26

**Authors:** Xiangchao Zhu, Yixiang Li, Le Luo, A. Ali Yanik

**Affiliations:** 1 Department of Electrical & Computer Engineering, University of California Santa Cruz, Santa Cruz, CA 95064, USA; 2 California Institute for Quantitative Biosciences (QB3), University of California Santa Cruz, Santa Cruz, CA 95064, USA

**Keywords:** optical chromatography, optofluidic, biosensing, label-free separation, plasmonic microlens, tunable resistive pulse sensing

## Abstract

Optical chromatography (OC) has emerged as a powerful, label-free technique for the precise manipulation and separation of micro- and nanoparticles based on their intrinsic biophysical properties, including size, refractive index, and morphology. By balancing optical radiation pressure with fluid drag forces, OC enables high-resolution sorting of diverse analytes—from synthetic colloids to biological cells and pathogens—without the need for fluorescent labels or chemical modifications. Recent advancements in integrated optofluidic platforms, such as plasmonic microlens arrays, fiber-based systems, and hybrid optical–electrical detection approaches, have significantly enhanced OC capabilities, addressing long-standing challenges in scalability, throughput, and sensitivity, and facilitated its transition toward compact, application-oriented analytical platforms. These innovations have expanded OC applications in critical biomedical fields, including exosome isolation, pathogen detection, and viral infection monitoring. Furthermore, the integration of OC with tunable resistive pulse sensing (TRPS) presents a promising avenue for simultaneous particle fractionation and characterization, overcoming key limitations of conventional resistive pulse techniques. In this review, we provide a comprehensive overview of the fundamental principles of OC, followed by recent progress in particle separation strategies and integrated optofluidic system design. We further highlight emerging applications in bioanalysis and discuss future directions toward high-throughput, multimodal, and clinically relevant OC platforms.

## 1. Introduction

Light-mediated propulsive action, though imperceptible in daily life, has captivated mathematicians, astronomers, and physicists for centuries across scales ranging from the subatomic to the astronomical. The first experimental demonstration of optical radiation-pressure forces by Lebedev in the early 1900s marked the beginning of optical manipulation, which has since evolved into a cornerstone of modern science and engineering. The invention of lasers in the 1960s catalyzed a breakthrough with the development of optical tweezer technology by Arthur Ashkin, Steven Chu, and their collaborators at AT&T Bell Laboratories [1,2,3]. This milestone enabled the generation and precise control of strong optical trapping and propelling forces for mesoscopic and macroscopic systems using intense, collimated laser beams. Ashkin’s revolutionary work overturned the long-held belief, dating back to John H. Poynting in 1905, that optical forces were too minuscule to be of practical significance in practical applications [4,5].

Building on Ashkin’s pioneering contributions throughout the late 20th century [1,2,3,6,7,8,9,10], extensive research efforts have transformed optical manipulation from a nascent concept into a sophisticated and versatile scientific tool [11,12]. Over the past decades, the non-invasive, high-precision manipulation of matter via laser radiation has ushered in new eras in optics and photonics [13,14,15,16,17,18], atomic physics [19,20,21,22,23,24], optofluidics [25,26,27,28,29], levitodynamics (for spacecraft propulsion) [30,31], and biochemical [32,33,34,35,36,37], biophysical [38,39,40,41,42,43,44,45], and medical research [46,47,48,49]. Recent advances have further expanded the frontiers of optical manipulation, enabling investigations at the single-molecule level [50,51,52,53] and even surpassing the classical diffraction limit [54,55,56,57,58,59,60].

Among emerging applications, light-based chromatography technologies have garnered significant attention for their ability to perform in situ, contactless, non-destructive, and label-free micromanipulation and characterization of biotechnological samples [61,62,63,64,65,66,67]. Optical chromatography (OC), first conceptualized by Kaneta et al. [61], operates through the interplay of optical radiation pressure and fluid drag forces in an axial configuration [66,67,68,69]. As a flow-assisted, laser-based analytical technique, OC offers advantages over optical tweezers by minimizing radiation-induced thermal damage to biological specimens [70,71,72]. Leveraging advancements in micro-/nanofabrication from telecommunications, OC systems have evolved into compact, integrated platforms utilizing lab-on-a-chip microfluidic devices [63,64,65].

This burgeoning field holds promise for biomedical applications, including rapid, non-invasive separation of biological analytes such as viruses, bacteria, yeast, erythrocytes, and pollen [73,74]. OC is of particular interest to biodefense and public health agencies due to its potential for identifying biological threat agents [73,74,75] and detecting pathogens for infection control (e.g., sepsis) [76]. Recent successes include distinguishing closely related bacterial strains [76,77], label-free viral detection [78,79,80], and fractionating blood components like cells and extracellular vesicles [65,81,82,83,84,85]. Remarkably, OC has achieved differentiation of bioparticles with size differences as small as 70 nm [86] and single-gene-modified cells based on refractive index variations [87].

Here, this review provides a comprehensive overview of recent advances in optical chromatography (OC) and explores emerging applications, from label-free exosome sorting for theragnostics to rapid infectivity assays for vaccine development. Despite significant progress in OC, comprehensive review articles focusing specifically on this technique remain limited in recent years, with most studies instead incorporated within broader optofluidics and optical manipulation frameworks [88,89]. Rather than an exhaustive literature survey, this work aims to bridge optical chromatography with emerging optofluidic technologies, serving as a practical guide to current experimental OC systems while emphasizing high-efficiency, high-throughput separation and dynamic analysis of nano-/microscale objects, including blood components.

The review is structured as follows: Section 2 outlines the theoretical framework for understanding OC-based separation mechanisms, including experimentally validated models for calculating optical and hydrodynamic forces on particles under varying conditions. Section 3 examines OC-based sorting of polymer particles, biological cells, and pathogens by size, composition, shape, and other intrinsic properties. It also explores on-chip laser beam delivery, high-resolution nanoparticle separation, and multi-component mixture fractionation, alongside real-world applications such as biodefense. Section 4 introduces an integrated optofluidic nanoplasmonic chromatography technique designed to overcome current OC limitations. Finally, we present perspectives on future developments and potential applications of OC.

## 2. Theoretical Aspects of Optical Chromatography

**Figure 1:**
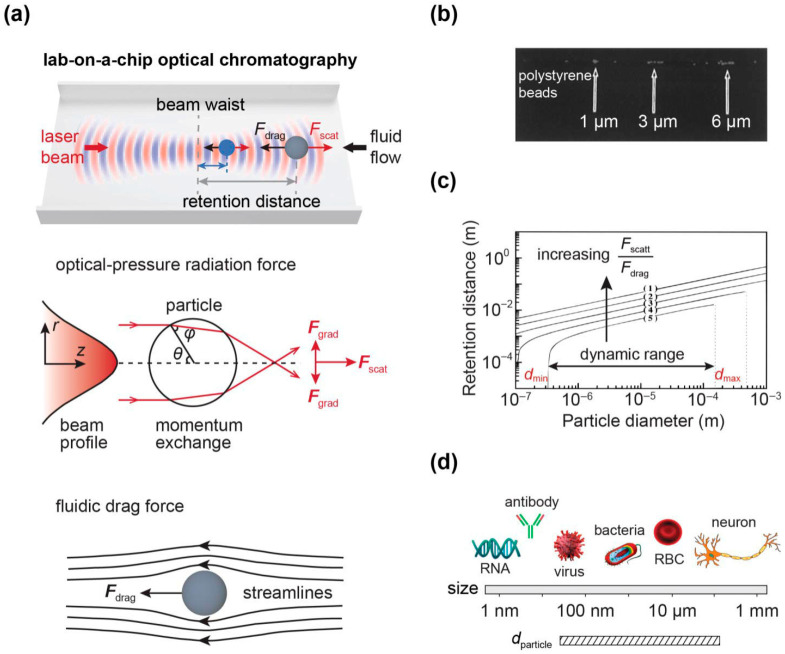
The fundamental components and operational principles of OC in a microfluidic environment. The top panel in (**a**) schematically illustrates the lab-on-a-chip OC configuration, featuring a mildly focused laser beam integrated with a counter-flow microfluidic system [67]. The middle panel details the momentum transfer mechanism between the laser photons and a suspended dielectric particle, while the bottom panel demonstrates the force balance between the propelling optical radiation pressure and opposing fluidic drag force that enables particle retention [63,67]. Experimental validation of the OC technique is shown in panel (**b**), where three distinct populations of polystyrene microspheres (1, 3, and 6 μm diameters) are spatially resolved along the laser axis, demonstrating the system’s size-based separation capability [67]. Panel (**c**) quantitatively illustrates how the retention distance—defined as the equilibrium position of particles relative to the laser focus—can be systematically modulated by varying the laser power to flow rate ratio (1) 10^6^, (2) 10^5.5^, (3) 10^5^, (4) 10^4.5^, and (5) 10^4^ W m s^−1^ [63]. The comprehensive performance characteristics of OC are summarized in panel (**d**), where the shaded bar represents the effective operational range for manipulating bioparticles of varying diameters *d*_particle_. This visualization highlights the technique’s broad dynamic range, spanning from nanoscale to microscale biological specimens [63,67]. Together, these panels demonstrate how optical chromatography combines precise optical forces with microfluidic control to achieve label-free, size-dependent separation of particles, with important applications in biological analysis and materials science [63,67].

In a typical OC system, a laser beam is mildly focused into a microfluidic channel containing dielectric particles suspended in a counter-flowing solution (Figure 1a). Unlike conventional optical trapping, which employs high-numerical-aperture (NA > 1.3) objectives for tight focusing, OC utilizes a long focal length lens (e.g., 75 mm) to achieve gentle beam confinement [90]. When a dielectric particle interacts with the laser beam, it experiences an optical scattering force (*F*_scatt_) due to momentum transfer from photons. Simultaneously, the fluid flow exerts a hydrodynamic drag force (*F*_drag_) opposing the laser propagation direction. The equilibrium position of the particle—where *F*_scatt_ and *F*_drag_ balance—depends on its size, enabling spatial separation of particles along the beam axis (Figure 1a). Particles of different sizes establish distinct equilibrium positions along the beam axis, resulting in different retention distances. As illustrated in Figure 1a, larger particles are retained further from the focal point due to stronger optical scattering forces, whereas smaller particles equilibrate closer to the beam waist. Thus, retention distance serves as a direct physical observable linking particle properties to separation behavior.

Kaneta et al. [63] first established the theoretical foundation for OC in 1997, modeling the interaction between a TEM_00_ laser mode and a dielectric particle larger than the wavelength of light but smaller than the beam waist (Figure 1b). Using ray optics, they derived *F_scatt_* by analyzing momentum exchange at the particle’s surface via Fresnel reflection and refraction. The scattering force is expressed as follows:

(1) $$F_{scatt}=\frac{2n_{1}P}{c}{\left(\frac{a}{\omega }\right)}^{2}Q^{*}$$

where *n*_1_ is the medium’s refractive index, *P* the laser power, *c* the speed of light, *a* the particle radius, and *ω* the beam radius. The beam radius varies with distance from the focal point (*z*) as described:

(2) $$\begin{array}{l} \omega^{2}=\omega_{0}^{2}\left[1+{\left(\frac{z}{z_{c}}\right)}^{2}\right]\\ z_{c}=\frac{\pi \omega_{0}^{2}}{\lambda } \end{array}$$

where *ω*_0_, *z*_c_ and *λ* denote the focal spot size, confocal distance, and laser wavelength, respectively.

The momentum transfer efficiency (Q^*^) is determined by integrating Fresnel coefficients [68]:

(3) Q*=∫0π2sin2θ Qθ dθ

which depend on the incidence angle (*θ*). By incorporating Snell’s law (*n*_1_sin*θ* = *n*_2_sin*φ,* where *n*_2_ is the particle’s refractive index and *φ* the refraction angle), *Q*(*θ*) can be expressed as:

(4) $$Q\left(\theta \right)=\frac{1}{2}\left\{1+R\cos2\theta -\frac{T^{2}\left[\cos\left(2\theta -2\varphi \right)+R\cos2\theta \right]}{1+R^{2}+2R\cos\varphi }\right\}$$

(5) $$R=\frac{1}{2}\left[\frac{\sin^{2}\left(\theta -\varphi \right)}{\sin^{2}\left(\theta +\varphi \right)}+\frac{\tan^{2}\left(\theta -\varphi \right)}{\tan^{2}\left(\theta +\varphi \right)}\right]$$

(6) $$T=\frac{1}{2}\left[\frac{\sin2\theta \;\sin\varphi }{\sin^{2}\left(\theta +\varphi \right)}+\frac{\sin2\theta \;\sin\varphi }{\sin^{2}\left(\theta +\varphi \right)\cos^{2}\left(\theta -\varphi \right)}\right]$$

Meanwhile, *F_drag_* follows Stokes’ law, scaling with particle radius *a*, fluid viscosity (*μ*), and dynamic viscosity of the fluid *η* as the particle moves at velocity (*ν*):

(7) $$F_{d}=6\pi \eta av$$

The net force (*F_net_*) acting on the particle governs its motion until equilibrium is reached, where *F_scatt_* = *F_drag_*.

(8) $$F_{net}=\frac{2n_{1}P}{c}{\left(\frac{a}{\omega }\right)}^{2}Q^{*}-6\pi \eta av$$

The retention distance (*z_r_*), defined as the equilibrium position’s offset from the laser focus, is derived to be:

(9) $$z_{r}=\frac{\pi \omega_{0}^{2}}{\lambda }\sqrt{\frac{n_{1}PQa}{3\pi \eta vc\omega_{0}^{2}}-1}$$

This distance depends on particle size, refractive index, laser power, and flow rate. Reducing the laser power shifts the particle toward the focus, and eventual elution occurs when *F_scatt_* is insufficient to counteract *F_drag_*. The minimum retainable particle diameter (*d_min_*)—where *z_r_* = 0—is given by:

(10) $$d_{\min}=2a_{\min}=\frac{3\pi \eta vc\omega_{0}^{2}}{n_{1}PQ^{*}}$$

which represents the threshold for optical retention at maximum *F_scatt_*.

Conversely, the maximum particle diameter (*d_max_*) arises when the beam waist is negligible compared to the particle size (*ω* ≪ *2a*). Here, momentum transfer becomes size-independent, and *F_scatt_* simplifies to:

(11) $$F_{scatt}=\frac{2n_{1}P}{c}Q\left(0\right)$$

where *Q*(0) is derived at normal incidence (*θ* = 0°). The expression for *d*_max_ follows in:

(12) $$d_{\max}=2a_{\max}=\frac{2n_{1}PQ\left(0\right)}{3\pi \eta vc}$$

The dynamic range of OC, defined as *d*_max_/*d*_min_, is critical for system characterization. Plotting *z_r_* against particle diameter (Figure 1c) reveals *d*_min_ and *d*_max_ at the curve’s intercepts with the diameter axis. Adjusting laser power and flow rate can extend this range significantly, enabling separation across scales from nanometers to micrometers (Figure 1d).

The equilibrium dynamics of optical chromatography are governed by the retention distance (*z_r_*), which balances optical and hydrodynamic forces. The system’s performance is determined by its size dependence, where *d*_min_ and *d*_max_ define the operational limits of particle manipulation. A key advantage of OC is its tunability, as the dynamic range can be expanded by increasing the laser power-to-flow rate ratio. This theoretical framework provides a foundation for optimizing OC systems for diverse applications, ranging from nanoparticle sorting to microscale bioparticle analysis.

## 3. Label-Free OC for Micro-/Nanoparticle Analysis

OC has emerged as a powerful and versatile technique for the precise characterization and separation of microscopic particles, leveraging the delicate balance between optical radiation forces and fluid drag in an axial configuration [63,64,91,92,93,94,95,96]. Unlike conventional bulk-sample methods, OC enables label-free, contactless, and non-invasive manipulation of both single particles and complex suspensions within integrated lab-on-a-chip systems [61,97]. This capability makes it particularly valuable for analyzing biological samples and for broader applications in biomedical diagnostics [98], where it can process complex matrices such as blood and detect pathogens without requiring fluorescent labels, antibodies, or prior knowledge of sample composition—all while maintaining high sensitivity, selectivity, and cost-effectiveness [64,76,78,98].

Compared to traditional chromatography techniques like size exclusion or hydrodynamic chromatography [99], OC offers a significantly broader dynamic range, capable of resolving particles with diameters spanning from ~1 μm to 100 μm. By adjusting the laser power and flow rate, this range can be extended over four orders of magnitude under optimized conditions [63]. The technique’s flexibility has enabled its application across diverse research areas, including the separation of polymer particles, aerosol droplets, blood cells, and pathogens based on size [70,86,97,100,101,102,103,104,105,106,107,108,109,110], refractive index [62,69,90,106,111,112,113,114,115], shape and morphology [65,77,116,117,118], and hydrodynamic properties [77,119,120,121]. Beyond particle sorting, OC has been employed to study biomolecular interactions, such as measuring antigen–antibody binding kinetics [122,123,124], assessing cellular mechanical properties [125,126], and characterizing viral infections [78,79,80].

These capabilities highlight OC’s potential as a transformative tool in fields ranging from materials science to biomedical diagnostics, offering a unique combination of high precision, label-free operation, and compatibility with miniaturized analytical platforms.

### 3.1. Selective Separation Based on Intrinsic Biophysical Properties

**Figure 2:**
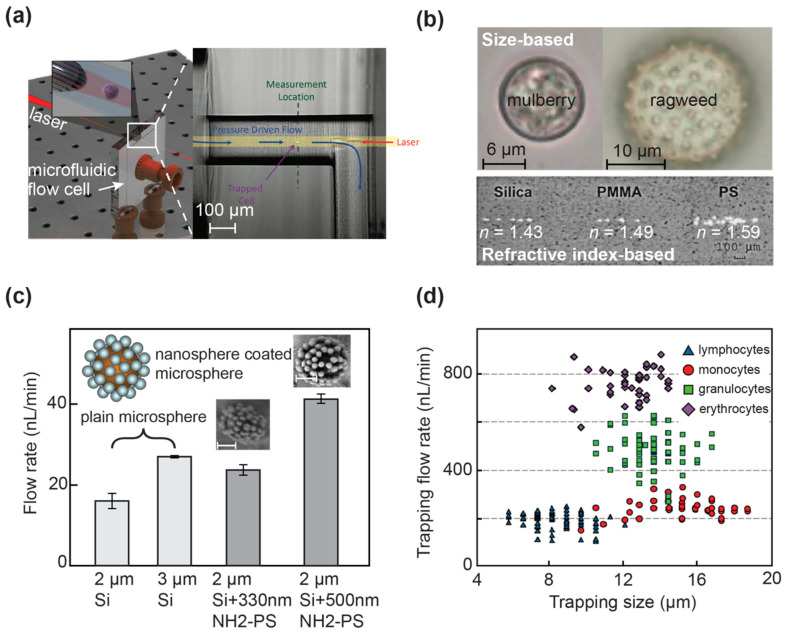
Key experimental configurations and results that demonstrate the capabilities of OC for particle separation and analysis. Panel (**a**) presents the microfluidic flow cell used for OC separations, showing both the complete device with fluidic connections (left) and a magnified view of the separation region (right) [64]. The blue arrow indicates the sample flow direction, while the red arrow marks the propagation direction of the focused laser beam that enables the optical separation mechanism. The technique’s application to biological samples is demonstrated in panel (**b**), which shows microscope images of successfully separated mulberry and ragweed pollen at 25× magnification [69]. This visual evidence confirms OC’s ability to discriminate between different biological particles based on their physical properties. Panel (**c**) presents quantitative data highlighting the system’s sensitivity to both chemical composition and size differences. The bar graph compares the separation efficiency for particles of identical size but different surface coatings (varying refractive index) as well as particles with subtle size variations but identical chemical composition. These results underscore the technique’s dual capability for chemical and physical characterization. The clinical potential of OC is showcased in panel (**d**) through the successful discrimination of major blood components [64]. The scatter plot correlates trapping flow rates with cell sizes for lymphocytes, monocytes, granulocytes, and erythrocytes, demonstrating the method’s ability to characterize complex biological samples without labels or markers. Collectively, these panels demonstrate the versatility of OC across different applications, from fundamental studies of particle-light interactions to practical biomedical analyses. The visual evidence supports the technique’s capabilities for sensitive, label-free separation and characterization of diverse particulate systems. (**a**,**d**) Reproduced with permission from [64], Copyright 2011, Analytical Chemistry. (**b**) Adapted from [62,69]. (**c**) Adapted from [118].

The remarkable selectivity of OC stems from its ability to discriminate particles based on their intrinsic biophysical properties, including size, shape, refractive index, surface roughness, and cellular structure [64]. In a standard experimental configuration (Figure 2a), a mixture of micron-scale particles or cells flows through a microfluidic channel counter to a loosely focused laser beam aligned along the channel axis. When exposed to this optical field, particles experience photon momentum transfer that creates a size- and composition-dependent retardation force, effectively slowing their movement through the channel [61,63,75]. This precise equilibrium between optical radiation pressure and hydrodynamic drag forces enables reproducible separation of complex particle mixtures based on their fundamental physical characteristics.

The separation mechanism follows a well-defined physical relationship (Equation (9)), where the equilibrium retention position relative to the beam waist increases proportionally with both particle size and refractive index. Larger particles or those with higher refractive indices experience greater optical forces and are consequently displaced further from the focal point [61,63,75]. This principle allows for spatial fractionation of multicomponent samples, including inorganic, polymeric, and biological particles, which can be collected at distinct positions along the microchannel [62,75,97,100,102].

A particularly effective elution strategy involves gradual reduction in laser power, enabling sequential release of particles based on their physical properties [103]. As the optical force diminishes, the smallest particles or those with lowest refractive indices are first to traverse the beam waist and exit the separation region. Continued power reduction then releases progressively larger or higher-refractive-index particles in a size- or composition-sorted manner. This active elution approach not only facilitates precise on-chip manipulation [75] but also enables straightforward determination of particle characteristics (dimension and refractive index) through their elution profiles [103]. The method’s ability to correlate elution order with physical properties makes it particularly valuable for analytical applications requiring characterization of unknown samples.

The capabilities of OC for size- [61,63,69,74,86] and composition- [62,69,90,108] based particle separation have been extensively validated by multiple research groups. Hart and colleagues made particularly noteworthy contributions, demonstrating millimeter-scale separation of biological particles including paper mulberry (12 μm) and ragweed (28 μm) pollen at remarkably high flow velocities of 100 μm/s [69]. Their work extended beyond size-based separation to include discrimination of colloidal polymer microparticles with nearly identical sizes but subtle refractive index differences below 0.06 [62] (Figure 2b).

The sensitivity of OC techniques has been progressively refined, with recent studies achieving resolution of nanometer-scale size differences down to 70 nm for both polymeric and inorganic particles of identical chemical composition [86,108]. This exceptional resolution highlights the technique’s potential for sophisticated analytical applications. In addition, Darmawan et al. demonstrated that mid-infrared optical force chromatography can provide wavelength-selective separation based on molecular vibrational absorption, thereby offering an additional level of selectivity beyond conventional refractive index contrast [127].

Transitioning from fundamental studies to practical implementations, researchers have employed soft-lithography techniques [128] to fabricate optimized microfluidic networks that provide precisely controlled flow environments [90]. Using these advanced systems with a low-power green laser (515 nm), the team successfully demonstrated clinically relevant separations, including the isolation of *Bacillus anthracis* (*B.a.*) spores—the causative agent of anthrax—from environmental pollen contaminants [90]. This achievement holds particular significance for biodefense applications, given *B.a.* status as a potential biological warfare agent, and showcases OC’s potential for real-world security and diagnostic applications.

The progressive refinement of OC systems, from fundamental particle studies to practical biological separations, demonstrates the technique’s evolving capabilities and expanding range of applications in both scientific research and security-related fields. These advances underscore OC’s unique advantages for sensitive, label-free particle analysis in complex matrices.

### 3.2. Expanding the Capabilities of OC: Surface Morphology and Biological Applications

Beyond size and refractive index-based separations, OC has demonstrated remarkable sensitivity to subtle differences in particle surface morphology. Taylor and colleagues developed an innovative approach using bio-inspired cellular models to investigate how surface roughness influences light–matter interactions in OC systems [118]. Through an amine-aldehyde assembly process [129], they created composite particles by conjugating glutaraldehyde-activated silica microspheres with amine-functionalized polystyrene nanospheres. These engineered particles exhibited nearly double the optical force compared to smooth silica microspheres of equivalent size (Figure 2c), a phenomenon attributed to enhanced light scattering by the nanoscale surface features.

This fundamental understanding enabled the team to achieve separation of closely related Bacillus spores—*B.a.* and *Bacillus thuringiensis* (*B.t.*)—which differ in their exosporium morphology [77]. The separation process was further influenced by shape differences, with *B.t.* spores displaying a more pronounced oblate spheroid shape and larger exosporium compared to *B.a.* These morphological variations affected both optical forces and fluidic drag, contributing to distinct retention positions in the OC system.

Building on these principles, Hebert’s group made a significant breakthrough by demonstrating the first label-free discrimination of human blood components, including erythrocytes, monocytes, granulocytes, and lymphocytes [64] (Figure 2d). This work capitalized on the inherent differences in optical pressure resulting from variations in cellular size, shape, refractive index, and morphology. The successful characterization of blood constituents represents a major step toward developing practical lab-on-a-chip devices for clinical applications, particularly for early detection of blood-borne pathogens and disease prevention.

These advances highlight OC’s evolving capabilities beyond simple particle sorting, demonstrating its potential for sophisticated biological analyses. The technique’s sensitivity to nanoscale surface features and ability to discriminate between closely related biological particles position it as a powerful tool for both fundamental research and practical diagnostic applications.

### 3.3. Recent Advances in Integrated Optical Chromatography Platforms

**Figure 3:**
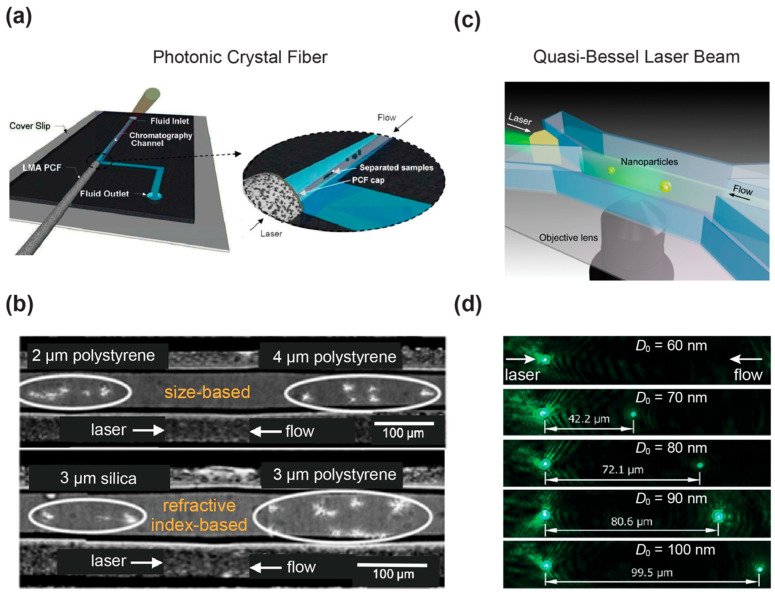
Two innovative approaches for integrated OC platforms that enhance performance while reducing system complexity. Panel (**a**) illustrates the design of a photonic crystal fiber (PCF)-based OC chip that enables direct on-chip beam delivery into the microfluidic channel. This compact configuration supports multi-wavelength operation, allowing simultaneous OC separation, fluorescence excitation, and real-time monitoring of particle dynamics within a single integrated platform. The system’s capabilities are demonstrated in panel (**b**), which presents results for both size-based separation (2 μm vs. 4 μm particles) and refractive-index-based separation (polystyrene vs. silica particles). An alternative high-resolution approach is depicted in panel (**c**), featuring a quasi-Bessel beam OC chip design. This configuration generates an extended focus beam with unique propagation characteristics ideal for precise particle manipulation. As shown in panel (**d**), the system achieves exceptional separation resolution, successfully distinguishing nanoparticles with diameter differences as small as 10 nm under identical operating conditions. These technological advances demonstrate the potential for OC systems to perform sophisticated separations in compact, integrated formats suitable for both research and clinical applications. (**a**,**b**) Reproduced from [111] under a Creative Commons Attribution (CC BY) license. (**c**,**d**) Adapted from [107].

Recent advances in optical chromatography (OC) platforms can be broadly categorized into several key technological directions aimed at improving system integration, capabilities and practicality. These include (1) optical integration strategies that replace conventional free-space beam delivery with fiber- or chip-based systems, (2) beam engineering approaches that enhance optical force profiles and enable higher-resolution particle manipulation, and (3) microfluidic innovations that facilitate multi-stage separation and increased throughput. Collectively, these developments address longstanding challenges in system complexity, alignment sensitivity, and scalability, and have significantly expanded the practical applicability of OC. Representative examples from each category are discussed below. Ashok et al. developed a breakthrough integrated OC platform by combining a large mode area photonic crystal fiber (PCF) with a microfluidic channel [111]. This novel on-chip design eliminates the need for bulky free-space optics by enabling direct laser coupling through the PCF (Figure 3a). The system leverages the endlessly single-mode property of PCFs to support multi-wavelength operation, allowing simultaneous OC separation, fluorescence excitation, and real-time monitoring within a single compact device. The platform successfully demonstrated both size-based separation of 2 μm and 4 μm colloidal spheres and refractive-index-based separation of equivalently sized polystyrene and silica particles (Figure 3b) [111]. While this integration represents a major step toward portable OC systems, the high manufacturing cost of LMA PCFs may currently limit widespread adoption. Similarly, Milark et al. recently developed a 3D-printed lab-on-a-chip platform incorporating fiber-based beam delivery, enabling compact and accessible OC and sorting systems [130].

An alternative fiber-based approach was pioneered by Shi et al., who developed a PDMS-based optofluidic chip incorporating a micro-quadrangular lens [107]. Fabricated using standard soft-lithography processes, this platform transforms a green fiber laser into a quasi-Bessel beam with exceptional focusing properties (NA ~ 0.04, focus < 0.5 μm) and extended propagation length (>140 μm) (Figure 3c). These characteristics enabled the first demonstration of high-resolution nanoparticle separation, successfully distinguishing particles between 60 and 100 nm with 10 nm resolution (Figure 3d) [107], representing a significant advancement for nanoscale biomedical applications.

Despite these advancements, many OC platforms remain limited by alignment requirements across multiple domains. In addition to precise optical alignment between the excitation beam and the microfluidic channel, consistent particle positioning within the flow is also essential for reliable separation. Microfluidic strategies such as hydrodynamic focusing help confine particles into well-defined trajectories, addressing alignment in the fluidic domain. However, optical alignment in existing systems—particularly in partially integrated platforms such as fiber-based configurations—still relies on careful positioning of optical components, limiting robustness and scalability. To overcome these limitations, recent efforts have focused on self-aligned and fully integrated platforms that minimize external alignment requirements. These approaches, including waveguide-based and optical plasmonic platforms, are discussed in Section 4.

### 3.4. Microfluidic Innovations for Enhanced OC Performance

**Figure 4:**
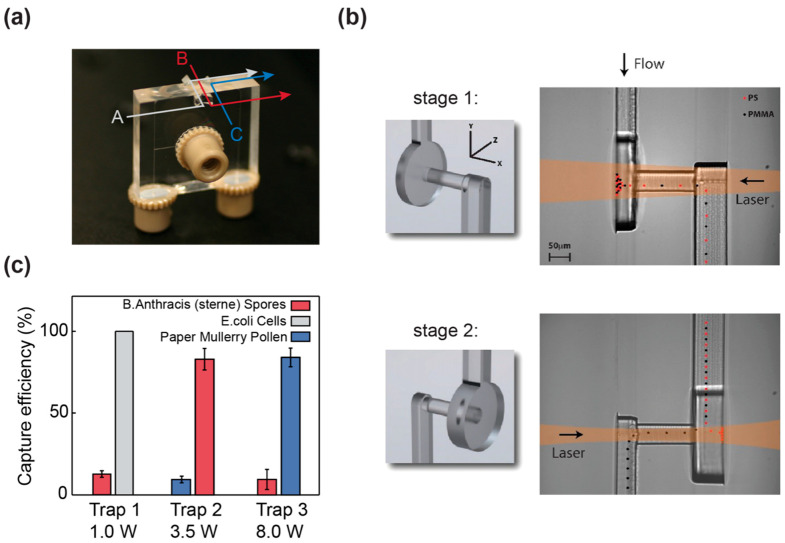
An advanced cascade OC system designed for enhanced separation of complex biological samples. Panel (**a**) shows the innovative flow cell architecture that enables multi-stage particle processing. The system employs a sophisticated two-stage mechanism illustrated in panel (**b**), combining initial hydrodynamic focusing with subsequent OC-based separation. This dual approach first aligns particles into a tightly focused stream before applying size- and refractive-index-dependent optical forces for precise fractionation. The system’s performance is demonstrated in panel (**c**), which documents the successful separation of a challenging biological mixture containing paper mulberry pollen, *Bacillus anthracis* (*B.a.*) spores, and *Escherichia coli* cells. This achievement represents a significant advancement over conventional OC systems, as it enables simultaneous processing of multiple particle populations within a single integrated platform. The cascade design particularly excels at handling heterogeneous biological samples, offering improved resolution and throughput compared to traditional single-stage separation methods. These developments highlight the ongoing evolution of OC technology toward more sophisticated analytical capabilities for complex real-world samples. (**a**,**b**) Reproduced with permission from [100], Copyright 2009, American Institute of Physics. (**c**) Adapted from [102].

Substantial progress has also been made in microfluidic design to improve OC separation capabilities. The Hart group developed an innovative glass-based flow cell supporting three independent laser separation regions [81,100,102]. This system implements a two-stage separation process (Figure 4a,b): first, hydrodynamic focusing aligns particles into single file, followed by size- and refractive-index-based separation in the OC region. The design enables simultaneous concentration of separated particles against the channel walls, representing a major improvement over conventional single-separation OC chips. Using this approach, the team achieved efficient fractionation of complex biological mixtures containing paper mulberry pollen, *B.a.* Sterne strain spores, and *E. coli* cells (Figure 4c) [81,100,102], demonstrating significant potential for processing heterogeneous biological samples.

These technological advances—encompassing both optical integration and microfluidic innovation—have collectively expanded OC’s capabilities from fundamental research toward practical analytical and biomedical applications. The development of compact, high-resolution systems with multi-component separation capacity addresses critical needs in fields ranging from nanomedicine to pathogen detection.

### 3.5. OC as a Bioanalytical Sensing Platform

**Figure 5:**
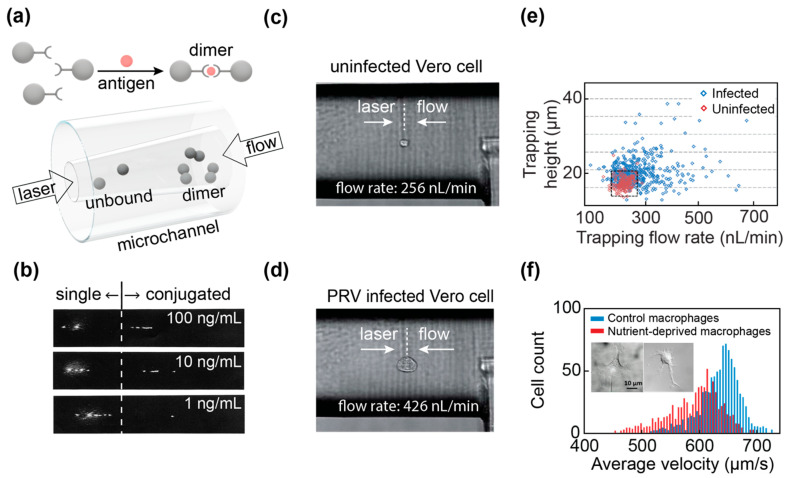
The diverse biomedical applications of OC through several key experimental demonstrations. Panel (**a**) illustrates the conceptual framework for applying OC to immunoassays, where antibody-functionalized microspheres enable antigen detection. The technique′s analytical capabilities are shown in panel (**b**), where a series of images documents the separation of single and conjugated immunobeads, allowing quantitative measurement of antigen concentrations. The utility of OC for virology studies is presented in panels (**c**,**d**), comparing microscope images of uninfected versus pseudorabies virus (PRV)-infected Vero. These visual results correlate with measurable biophysical changes induced by viral infection. Panel (**e**) extends this analysis through neural network classification, clearly distinguishing infected (blue) from uninfected (red) cell populations based on their optical characteristics. The final panel (**f**) showcases the advanced capabilities of flow-through OC, demonstrating the separation of nutrient-deprived macrophages (red) from control cells (blue) based on velocity profiles. This application highlights the technique′s ability to detect subtle physiological changes in response to environmental stimuli. Collectively, these examples demonstrate OC’s versatility across multiple biomedical applications, from molecular detection to cellular analysis. (**a**) Adapted from [122]. (**b**) Adapted from [124]. (**c**–**e**) Reproduced with permission from [78] Copyright 2014, Royal Society of Chemistry. (**f**) Adapted from [65].

The emerging potential of OC for biomedical applications has generated significant research interest in recent years [65,76,78,79,122,124]. The Imasaka group pioneered the biological application of OC by developing an immunoassay platform capable of monitoring immunological reactions in real time and detecting single molecules at clinically relevant concentrations [122,124]. Their innovative approach utilized antibody-functionalized polymer microspheres that formed dimer complexes upon antigen recognition (Figure 5a). These conjugated microspheres exhibited up to 500 μm greater retention distance compared to unbound particles due to increased optical radiation pressure. Through CCD imaging and quantitative analysis of free versus bound microspheres, the system achieved sensitive detection of antigen concentrations below 1 ng/mL while simultaneously monitoring immunocomplex kinetics (Figure 5b) [122,124].

### 3.6. OC for Viral Infection Analysis and Vaccine Development

Recent advancements in OC have enabled the development of analytical optical force measurement techniques for label-free identification of viral infections, offering significant potential to accelerate vaccine development and manufacturing [78,79]. This approach relies on establishing an equilibrium state within an optical microflume, where individual particles or cells are held at a fixed position by precisely balancing optical forces with controlled fluid flow rates [64,78]. The underlying principle exploits the direct proportionality between optical force and trapping flow rate, allowing straightforward quantification of forces exerted on biological samples [63,86].

Hebert and colleagues applied this method to study pseudorabies virus (PRV) and vesicular stomatitis virus (VSV) infections in Vero cells—a critical cell line for human vaccine production [78,79]. Their experiments revealed distinct biophysical changes in infected cells, including increased size, enhanced transparency, and altered optical trapping behavior. While uninfected Vero cells were stably trapped at 256 nL/min (Figure 5c), PRV-infected cells required a significantly higher flow rate of 426 nL/min to maintain position (Figure 5d). The researchers attributed these changes to two key mechanisms: (1) virus-induced syncytium formation, leading to cell fusion and altered morphology [131], and (2) accumulation of high-refractive-index viral proteins, which modify the cells’ optical properties [132,133].

To automate classification, the team employed a neural network-based pattern recognition system (MATLAB′s Neural Network Analysis toolbox), achieving highly accurate discrimination between infected and uninfected cell populations based on their optical force signatures (Figure 5e). This label-free approach provides a rapid and sensitive method for monitoring viral infectivity, offering valuable insights throughout the vaccine development pipeline—from early-stage research to manufacturing quality control. The technique’s ability to detect subtle biophysical changes in infected cells without requiring fluorescent labels or antibodies makes it particularly attractive for high-throughput applications in virology and vaccine development.

### 3.7. Flow-Through OC for High-Throughput Cell Analysis

A significant advancement in OC was achieved by Lu et al. through the development of flow-through OC (FT-OC), an innovative approach that enhances the analytical capabilities of traditional static OC methods [65]. Unlike static OC, which requires precise flow rate adjustments to trap particles or cells at fixed positions, FT-OC operates by maintaining a constant laser power while allowing samples to continuously pass through a detection window at a predetermined flow rate. This continuous-flow design enables real-time, on-the-fly measurement of key biophysical parameters—including velocity, shape, and morphology—at single-particle or single-cell resolution.

The major advantage of FT-OC lies in its dramatically improved throughput, capable of analyzing hundreds of particles or cells per minute, far exceeding the capacity of static OC systems [65]. The researchers validated the technique through two key demonstrations: (1) rapid, size-based separation of polymer microspheres, and (2) sensitive detection of autophagy-induced changes in live macrophages subjected to nutrient deprivation. Their findings revealed that nutrient-deprived macrophages exhibited a significant reduction in average velocity accompanied by a modest increase in cell size (Figure 5f). These changes reflect complex cellular responses to stress, including plasma membrane remodeling, cytoskeletal reorganization, and alterations in cytochrome redox states [134,135,136,137]. Collectively, these modifications increase the cells’ resistance to optical forces, resulting in slower transit through the detection window.

The FT-OC platform represents a major step forward in label-free cell analysis, combining high-throughput capabilities with sensitive detection of subtle physiological changes. By eliminating the need for batch-wise analysis, this approach opens new possibilities for real-time monitoring of cellular responses to environmental stimuli, drug treatments, or other perturbations. The technique’s ability to resolve population shifts without fluorescent labeling makes it particularly valuable for applications in cell biology, immunology, and drug discovery where non-invasive, high-content analysis is essential.

## 4. Self-Aligned and Fully Integrated Optical Chromatography Platforms

To improve the practicality and scalability of optical chromatography (OC), recent efforts have focused on the development of self-aligned and fully integrated platforms that reduce reliance on external optical alignment. These approaches leverage guided optical structures and on-chip integration to achieve more robust, compact, and user-friendly systems. In this section, we highlight emerging strategies toward alignment-resilient OC platforms. We first outline the key challenges limiting current implementations, followed by recent advances in waveguide-based systems and optical plasmonic platforms.

### 4.1. Challenges and Limitations in OC Implementation

Despite demonstrating exceptional separation efficiency, particularly when combined with multi-stage fractionation and network-based microfluidic purification systems [100,102], OC faces several significant barriers to widespread adoption [84,109]. The technique currently requires expensive laser systems to generate the necessary optical radiation-pressure forces within microfluidic channels [64], creating substantial cost barriers for many potential users. Furthermore, the optical setup presents technical challenges, as precise coupling of the laser beam into chromatography channels demands complex free-space alignment procedures involving multiple off-chip, bulky multi-axis positioners [63,111]. This requirement for specialist expertise and delicate optical adjustments complicates experimental procedures and limits reproducibility.

Most critically, the dependence on alignment-sensitive optical components, and high-cost laser systems creates major obstacles for scaling OC platforms to achieve multiplexed, high-throughput operation. These technical constraints currently prevent the translation of OC’s impressive analytical capabilities into practical, widely accessible systems. While recent advances in integrated optical designs show promise for addressing some of these limitations, significant engineering challenges remain before OC can achieve broad implementation across research and clinical settings.

### 4.2. Waveguide-Based Optical Chromatography Platforms

Waveguide-based optofluidic platforms provide a natural pathway for implementing optical chromatography (OC) in integrated formats, as they enable confined optical fields and extended light–matter interaction lengths within microfluidic channels. These features are directly aligned with the fundamental requirements of OC, where continuous optical forces govern particle transport and separation along the flow direction. From a theoretical perspective, optical forces and particle dynamics in waveguide geometries have been systematically studied, demonstrating that guided optical modes can support controlled particle motion and size-dependent transport under radiation pressure [138]. Collectively, these findings highlight the strong potential of waveguide-based systems for realizing compact, on-chip OC functionalities.

**Figure 6:**
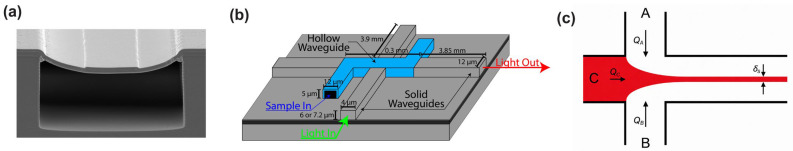
Waveguide-based optical and fluidic alignment strategies for integrated optical chromatography platforms. Panel (**a**): SEM images of hollow- and solid-core ARROWs. Reproduced from [140] under a Creative Commons Attribution (CC BY) license. The schematic in panel (**b**) illustrates the basic ARROW biosensor design. Reproduced from [142] under a Creative Commons Attribution (CC BY) license. (**c**) shows the schematic view of hydrodynamic focusing in a four-channel intersection. Reproduced from [144] under a Creative Commons Attribution (CC BY) license.

Building on this foundation, waveguide-integrated optofluidic platforms have been developed to enable on-chip light delivery and particle manipulation within microfluidic environments. Early implementations based on anti-resonant reflecting optical waveguides (ARROWs) by A.R. Hawkins and H. Schmidt, demonstrated the integration of solid-core and liquid-core waveguides on planar substrates, providing a versatile framework for light–matter interaction and optical sensing (Figure 6a) [139,140,141]. These platforms have been further extended to lab-on-a-chip systems, where guided optical modes enhance detection sensitivity and improve signal-to-noise performance (Figure 6b) [142]. In addition to optical confinement, effective OC implementation also requires precise control of particle positioning within the fluidic channel. Hydrodynamic focusing, a widely used microfluidic technique, leverages laminar flow to confine particles into narrow, well-defined trajectories (Figure 6c), thereby achieving reliable alignment in the fluidic domain. When combined with waveguide-based optical confinement, this dual-alignment strategy enables simultaneous control of both particle position and optical force distribution [143]. Compared to conventional free-space OC systems, where optical alignment and flow conditions must be independently optimized, integrating fluidic and optical alignment within a unified platform significantly improves system stability, reproducibility, and scalability. This combined approach provides a robust foundation for developing practical and high-throughput OC technologies.

### 4.3. Breakthrough in Integrated Nano-Optofludic Chromatography Platforms

**Figure 7:**
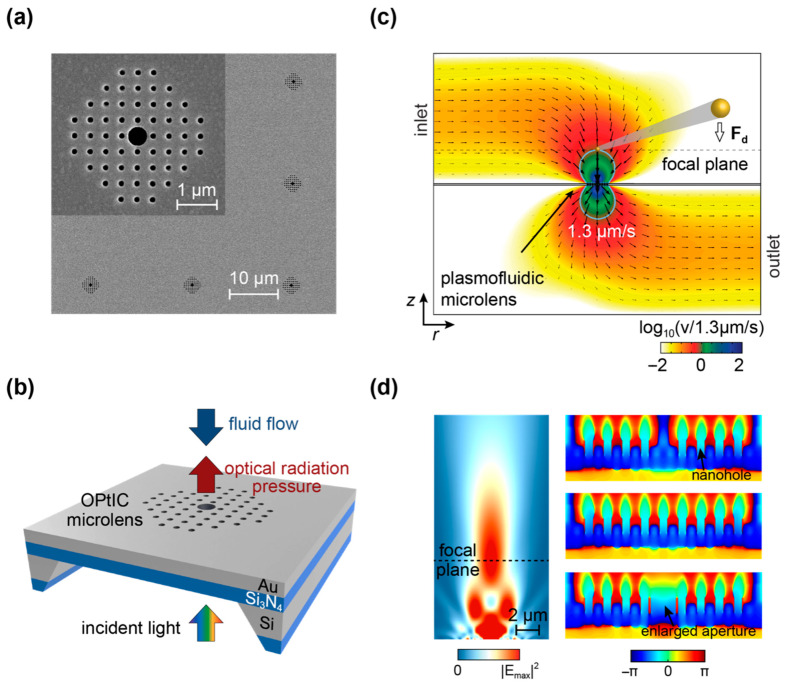
The innovative design and operational characteristics of the optofluidic plasmonic microlens platform for advanced optical chromatography applications. Panel (**a**) highlights the system’s planar architecture, enabling massive parallel integration with each microlens occupying a remarkably small footprint of approximately 16 μm^2^. The schematic in panel (**b**) illustrates the dual functionality of these microlenses, demonstrating both efficient light focusing through near-field phase modulation and controlled fluidic transport via a strategically enlarged central nanoaperture. The fluid dynamics performance is quantified in panel (**c**), showing the flow profile through a microlens with a focal point flow rate of *v*_0_ = 1.3 μm/s. Optical characterization results in panel (**d**) reveal the system’s exceptional focusing capabilities, with the left panel displaying the collimated beam profile under 633 nm illumination, and the right panel presenting finite-difference time-domain (FDTD) calculations of near-field phase distributions for varying central aperture diameters (0–500 nm). These computational and experimental results collectively demonstrate how the platform maintains excellent optical performance while achieving the necessary fluidic properties for chromatographic applications. The integrated design successfully addresses the fundamental challenges of traditional optical chromatography systems by combining precise optical manipulation with efficient microfluidic transport in a compact, scalable format. The platform’s ability to simultaneously control both optical and fluidic phenomena at sub-wavelength scales represents a significant advancement toward practical, high-performance lab-on-a-chip separation systems. Reproduced from [84] under a Creative Commons Attribution (CC BY) license.

Our research team has recently developed a novel monolithic OC platform that overcomes longstanding technical limitations through an innovative integration of nanoplasmonics and nanofluidics [84,109]. The system employs arrays of sub-wavelength Optofluidic PlasmonIC (OPtIC) microlenses (~200 nm thick), each comprising a 9 × 9 nanohole array (NHA) patterned in a free-standing silicon nitride membrane with gold coating (100 nm Au/5 nm Ti on 100 nm Si_3_N_4_) (Figure 7a,b). This compact 4 μm × 4 μm platform uniquely combines nanoplasmonic light manipulation with efficient nanofluidic transport through its periodic nanohole lattice [84,109].

The design features a strategically enlarged central aperture that serves multiple critical functions (Figure 7b,c): (1) enabling size-selective separation of exosomes through negative depletion; (2) reducing fluidic resistance by 100-fold for ultra-efficient flow; (3) achieving automatic self-alignment of optical and fluidic forces; and (4) maintaining exceptional light-focusing capabilities [84,109]. Optical characterization reveals the platform’s remarkable performance, with a 500 nm central aperture (surrounded by 150 nm nanoholes at 380 nm periodicity) focusing 633 nm unpolarized light to a tight 1.31 μm spot (Figure 7d, left panel) [84,109,145,146].

The underlying physics involves a plasmonic Talbot effect, where the finite 2D NHA creates self-imaging patterns through diffraction [84,109,145,146]. FDTD simulations confirm that varying the central aperture size (0–500 nm) minimally affects the characteristic checkerboard near-field intensity distribution (Figure 7d, right panel). Compared to conventional microlenses [147,148], our OPtIC design offers superior miniaturization, reduced chromatic aberration, and compatibility with high-intensity LED sources—making it ideally suited for label-free chromatographic applications. This breakthrough integration of optical and fluidic functionalities at the nanoscale represents a significant advance toward practical, high-performance OC systems.

### 4.4. Mechanisms of Particle Manipulation in Optofluidic Plasmonic Chromatography

**Figure 8:**
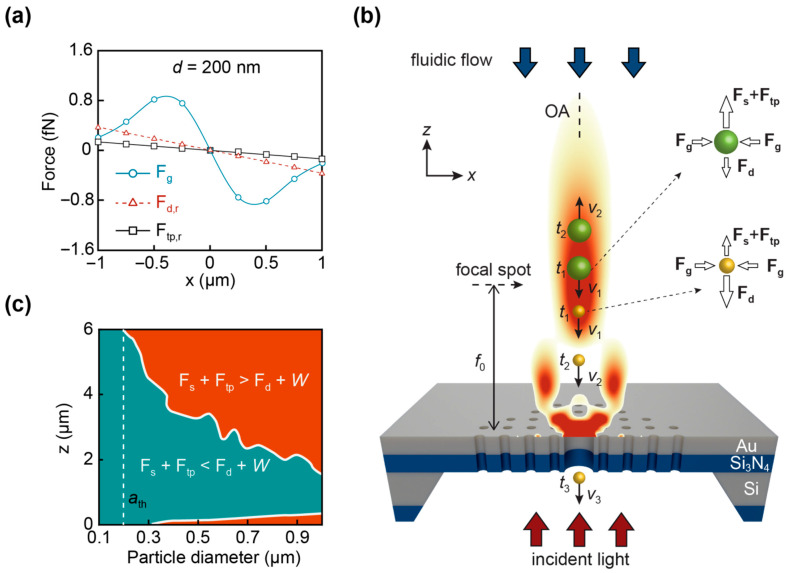
The fundamental force mechanisms enabling precise particle manipulation in the optofluidic plasmonic chromatography platform. Panel (**a**) demonstrates effective radial confinement of particles through the combined action of optical gradient (*F*_g_), radial drag (*F*_d,r_), and thermo-plasmonic convection (*F*_tp,r_) forces, which collectively minimize spatial dispersion and maintain particle alignment along the optical axis. The size-based separation principle is schematically depicted in panel (**b**), showing how differently sized particles respond to vertically aligned forces at the focal region. The platform’s unique design ensures automatic alignment of optical scattering (*F*_s_), fluidic drag (*F*_d_), and thermo-plasmonic convection (*F*_tp_) forces along the optical axis, enabling efficient sorting without complex external adjustments. Quantitative performance characteristics are presented in panel (**c**), showing the net force (*F*_net_ = *F*_s_ + *F*_d_ + *F*_tp_ + *W*) profiles for submicron particles (100 nm–1 μm) under standardized operating conditions (1.3 μm/s flow rate, 20 mW power at 633 nm wavelength). These results demonstrate the system’s ability to discriminate particles based on subtle size differences through carefully balanced force interactions, highlighting its potential for high-resolution separations in biomedical and analytical applications. Reproduced from [84] under a Creative Commons Attribution (CC BY) license.

The optofluidic plasmonic optical chromatography platform achieves precise particle control through a sophisticated balance of multiple forces acting on bioparticles [84,109]. During operation, particles experience three key radial forces that collectively focus them toward the optical axis (OA): optical gradient forces (*F***_g_**), radial drag forces (*F*_d,r_), and thermo-plasmonic convection forces (*F*_tp,r_). The thermo-plasmonic component arises from temperature gradients generated by electromagnetic heating of the OPtIC microlenses [84,109]. As demonstrated in Figure 8a for 200 nm nanoparticles, these combined forces effectively suppress spatial dispersion and maintain precise alignment along the OA (*x* = 0).

Along the vertical axis, particles are subject to competing forces: upward optical scattering (*F*_s_) and thermo-plasmonic convection (*F*_tp,r_) versus downward fluidic drag (*F*_d_) and gravitational (*W*) forces. The platform’s size-selective separation capability stems from careful control of these force balances, which vary with particle dimensions [84,109]. Figure 8b illustrates how this differential force balance enables complete particle separation—smaller particles experience a net downward force (*F*_net_ = *F*_s_ + *F*_tp_ − *F*_d_ − *W*) while larger particles are driven upward (*F*_net_ > 0).

Quantitative analysis of submicron particles (100 nm ≤ *d* ≤ 1.0 μm) with fixed density (1.05 g/cm^3^) and refractive index (1.55) reveals the system’s operational characteristics (Figure 8c). Calculations performed at 20 mW illumination (*λ* = 633 nm) and 1.3 μm/s flow velocity show distinct regimes: particles below 200 nm (left of vertical dashed line) are dominated by drag and gravitational forces (*F*_s_ + *F*_tp_ < *F*_d_ + *W*), following fluid streamlines through the central aperture for negative depletion isolation. Conversely, larger particles (*d* > 200 nm) experience stronger optical and thermal forces (*F*_s_ + *F*_tp_ > *F*_d_ + *W*), causing retention above the microlens [84,106].

The threshold separation size (*d*th) can be precisely tuned by adjusting illumination intensity and flow speed, demonstrating the platform’s flexibility for diverse separation applications. This multi-force control mechanism enables high-precision, size-based particle sorting while maintaining the benefits of compact integration and parallel processing inherent to the OPtIC microlens design.

## 5. Future Research Directions for OC

Optical chromatography (OC) has demonstrated strong potential for label-free particle separation and analysis; however, several challenges remain before it can be widely translated into practical applications. Future developments are expected to focus on improving system scalability, integration, and robustness while achieving more precise and reliable control of both optical and fluidic alignment.

Key directions include the development of high-throughput and scalable OC systems, as current implementations are often limited by single-channel operation and low processing rates. Approaches such as parallelized architectures, multiplexed microfluidic networks, and array-based designs may enable large-scale particle processing without compromising separation performance. In parallel, further integration and miniaturization—transitioning from free-space optics to waveguide-based and optofluidic platforms—will be critical for enhancing system stability and reducing alignment complexity, ultimately enabling portable and user-friendly OC devices.

In addition, integrating OC with complementary sensing modalities offers a promising route to enhance analytical capability. Hybrid systems combining optical chromatography with electrical, optical, or nanopore-based detection techniques can provide additional information such as particle size, mechanical properties, or molecular composition. For example, coupling OC with tunable resistive pulse sensing (TRPS) enables simultaneous separation and high-resolution characterization, facilitating more comprehensive analysis of heterogeneous particle populations. In addition to these directions, key challenges such as thermal effects from optical illumination, variability in biological samples, and fabrication complexity in integrated platforms must also be addressed to enable reliable and scalable OC systems.

### 5.1. Integration with Tunable Resistive Pulse Sensing (TRPS)

The continued advancement of OC technology points toward several promising research directions with significant potential for biomedical applications. One particularly compelling opportunity lies in the integration of OC with tunable resistive pulse sensing (TRPS) to create a unified analytical platform [149,150,151,152,153]. This hybrid approach would combine OC’s exceptional fractionation capabilities with TRPS’s sensitive detection and characterization functions, enabling comprehensive analysis of complex biological samples in a completely label-free manner.

TRPS technology operates on the well-established Coulter principle, providing simultaneous size profiling, identification, and quantification of submicron bioparticles including viruses, nucleotides, and exosomes without requiring chemical modifications [149,150,151,152,153]. The technique measures ionic current fluctuations as particles pass through a nanoscale aperture in a suspended membrane, generating characteristic electrical signatures for different particle types [154]. While TRPS has demonstrated remarkable potential for nanomedicine and bioanalytical applications, its widespread adoption faces a critical technical limitation: the persistent challenge of aperture blockage when analyzing heterogeneous biological samples [155,156].

This blockage issue proves especially problematic for clinically relevant extracellular vesicles like exosomes, which exhibit inherent size polydispersity and aggregation tendencies [157,158,159,160]. The integration with OC could provide an elegant solution to this limitation by performing pre-fractionation of complex samples prior to TRPS analysis. Such a combined system would not only mitigate aperture clogging issues but also enable correlated optical and electrical characterization of individual bioparticles, opening new possibilities for high-content, label-free biological analysis. This synergistic approach could significantly advance applications ranging from liquid biopsy development to pathogen detection and vaccine characterization.

### 5.2. Innovative Solutions for Aperture Blockage in TRPS Systems

Current approaches to address aperture blockage in tunable resistive pulse sensing (TRPS) systems involve intermittent measurement pauses for mechanical membrane agitation or stretching [161]. Existing mitigation strategies, such as intermittent membrane actuation or mechanical stretching, can temporarily alleviate clogging but often introduce increased operational complexity, reduced throughput, and potential variability in sensing performance.

**Figure 9:**
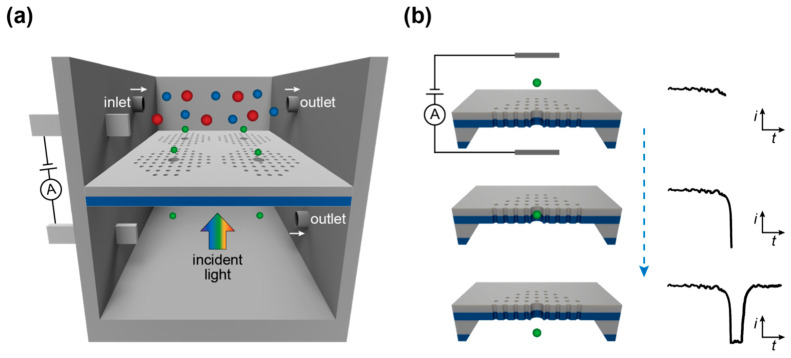
The innovative integration of optical chromatography with resistive pulse sensing in a unified platform. Panel (**a**) illustrates the compact optofluidic plasmonic microlens system capable of performing both label-free fractionation of complex particle mixtures and subsequent resistive pulse analysis of separated target particles. This combined approach enables comprehensive sample characterization within a single microfluidic device. The sensing mechanism is demonstrated in panel (**b**), which shows characteristic electrical current variations as separated particles translocate through the microlens structure. The symmetric pulse patterns reflect the passage of particles through cylindrical nanoapertures, providing simultaneous size and concentration information about the optically pre-sorted analytes. This integrated design overcomes traditional limitations of standalone resistive pulse sensing by combining precise optical manipulation with sensitive electrical detection.

One potential strategy is to involve the integration of OC or optofluidic structures with TRPS technology (Figure 9a) to enable pre-fractionation and controlled particle routing before electrical detection. By selectively manipulating particles based on their size and optical properties, such hybrid approaches can reduce the likelihood of clogging while improving measurement stability and reproducibility (Figure 9b). This synergistic approach addresses fundamental limitations of conventional TRPS while enabling new analytical possibilities for nanoscale biological characterization.

## 6. Conclusions

Optical chromatography (OC) has evolved from a specialized optical manipulation technique into a versatile analytical platform with broad potential across materials science and biomedicine. By leveraging the fundamental interplay between optical forces and fluid dynamics, OC enables high-resolution, label-free separation of particles and cells based on intrinsic biophysical properties—eliminating the need for fluorescent labels or chemical modifications. Recent innovations in integrated optofluidic designs, including plasmonic microlens arrays and hybrid optical–electrical detection systems, have addressed critical challenges in scalability, throughput, and sensitivity, facilitating the transition of OC toward more compact and application-oriented platforms. These developments highlight an increasing convergence between classical optical manipulation and modern optofluidic technologies, positioning OC as a bridge between fundamental light–matter interaction and integrated lab-on-a-chip systems.

Looking forward, further progress in miniaturization, multiplexing, and automation—together with the integration of complementary sensing modalities (e.g., resistive pulse sensing), data-driven analysis [162], and emerging optofluidic biosensing strategies [163]—will continue to expand the capabilities of OC for high-throughput and multimodal bioanalysis in clinical and point-of-care settings. As OC increasingly aligns with emerging optofluidic and applied biotechnologies, it holds strong promise for advancing diagnostics, drug development, and the understanding of cellular heterogeneity at the nanoscale.

## Data Availability

Not applicable. No new data were created or analyzed in this study. Data sharing is not applicable to this article.
